# Determinants of Cochlear Implant Evaluation Completion and Uptake in Children

**DOI:** 10.3390/jcm15072731

**Published:** 2026-04-04

**Authors:** Lisa R. Park, Shannon R. Culbertson, Dahvae Turner, Margaret E. Richter, Caitlin Sapp, Jennifer S. Woodard, Margaret T. Dillon

**Affiliations:** 1 Department of Otolaryngology/Head & Neck Surgery, University of North Carolina at Chapel Hill School of Medicine, Chapel Hill, NC 27514, USAmargaret_richter@med.unc.edu (M.E.R.); jennifer.woodard@unchealth.unc.edu (J.S.W.); mdillon@med.unc.edu (M.T.D.); 2 Department of Audiology, UNC Health, Chapel Hill, NC 27514, USA; shannon.culbertson@unchealth.unc.edu (S.R.C.); caitlin.sapp@unchealth.unc.edu (C.S.)

**Keywords:** cochlear implant, CI, pediatric, uptake, referral, children, traditional, non-traditional, candidacy, deaf, hearing loss, single-sided deafness

## Abstract

**Background/Objectives**: Approximately half of US children who meet traditional cochlear implant (CI) candidacy criteria receive an implant. As candidacy expands to include a broader range of hearing configurations, identifying factors that influence referral completion and CI uptake is increasingly important. This study examined the predictors of CI evaluation completion and surgery among children referred for CI assessment, including both traditional and non-traditional candidates. **Methods**: The medical records of pediatric patients referred for an initial CI evaluation from 2018 through 2024 were reviewed. Referral outcomes were categorized as evaluation not completed, candidate who declined surgery, or candidate who proceeded with surgery. Two binomial logistic regression models assessed the demographic, audiologic, and contextual predictors of CI evaluation completion and CI uptake, including age at referral, candidate type, insurance, referral year, communication mode, race/ethnicity, unaided thresholds, rurality, and county-level social health determinants. **Results**: The completion of the CI evaluation was significantly influenced by race/ethnicity, candidate type, referral year, and age. Mixed-race children demonstrated higher completion rates than White children. Completion was lower among children with single-sided deafness (SSD), children referred in 2022, and older children. Among children determined to be candidates, 69% proceeded with surgery. CI uptake showed similar patterns, with lower rates among Hispanic children, children with residual hearing or SSD, children referred in 2022, and older children. **Conclusions**: CI uptake at this center exceeded national averages but was associated with race/ethnicity, candidate type, age, and year of referral. Targeted counseling and outreach may improve timely referral and informed decision-making.

## 1. Introduction

Children with moderately severe or poorer levels of sensorineural hearing loss may obtain limited benefit from well-fit hearing aids, particularly when auditory access is insufficient to support the development of listening and spoken language. In such cases, a cochlear implant (CI) may provide improved access to sound. When limited aided benefit is suspected, children should be referred for a CI evaluation [1], during which candidacy is determined through interdisciplinary assessment that may include audiologic testing, aided speech recognition measures, speech and language evaluation, medical assessment, and the imaging of auditory anatomy. After evaluation, the families of CI candidates are tasked with using the information provided by the clinical team to decide whether to proceed with cochlear implantation.

Pediatric CI candidacy criteria have expanded substantially in recent years. In 2019, the US Food and Drug Administration (FDA) approved device labeling that expanded pediatric CI candidacy criteria to include those with asymmetric hearing loss (e.g., single-sided deafness). In 2025, the FDA approved labeling expanded further to include children as young as 7 months with bilateral profound sensorineural hearing loss and children 12 months and older with moderate-to-severe sensorineural hearing loss [2]. Multiple studies have demonstrated that pediatric patients outside traditional bilateral severe-to-profound candidacy criteria experience meaningful benefit from CI use, supporting this evolution of candidacy criteria [3,4,5,6,7,8,9].

As pediatric candidacy criteria expand, referral guidelines have shifted to better identify children who may benefit from a CI. Evidence-based recommendations encourage referral for CI evaluation when either ear meets any of the following: a four-frequency pure-tone average (0.5, 1, 2, and 4 kHz; 4FPTA) of ≥60 dB HL, ≤60% aided word recognition, or aided SII ≤ 60 [1,10,11,12,13]. However, referral alone does not ensure access to a CI. Families must first complete the candidacy evaluation and, if eligible, decide whether to proceed with implantation. These decisions may be influenced by a range of clinical and non-clinical factors.

For pediatric CI candidates, older age at referral, insurance type, socioeconomic vulnerability, and race have been linked to delays in implantation [14,15,16,17,18,19,20]. Children considered non-traditional CI candidates have historically been less likely to be referred for CI evaluation [10,21], and parental hesitancy is common for children with residual hearing [22,23]. However, most studies emphasize delays to surgery rather than earlier decision points in the referral pathway. Quantitative data examining CI uptake, defined as completion of the CI evaluation and proceeding with cochlear implantation among candidates, remain limited in pediatric populations.

Established barriers to hearing healthcare access may influence completing the CI evaluation and/or proceeding with cochlear implantation. One of the more consistent barriers to hearing healthcare is insurance source [24]. Compared with privately insured peers, children with Medicaid undergo implantation at later ages, face longer delays, and are less likely to be referred for CI evaluation [10,17,18,21,25,26]. Despite this, few studies examine insurance as a quantitative factor in families’ decisions to complete evaluation or proceed with implantation after referral.

Racial disparities are also documented. Children of color are less likely to be referred for CI evaluation [10] and less likely to follow through with CI recommendations than White children [25,26,27,28,29,30]. Age is associated with a reduced likelihood of referral and implantation [10,27], though pediatric-specific uptake data are sparse. Geographic disparities further constrain access. Families in rural areas travel farther and have fewer specialty services. Many parents in these areas have never even heard of a CI [28]. Adult studies link rural residence and social deprivation to reduced adherence to CI evaluation and follow-up [31], but pediatric data are limited. This gap is particularly relevant in states with large rural populations, such as North Carolina (NC).

Beyond insurance, race, age, and geography, broader socioeconomic vulnerability is associated with barriers to care and delayed implantation in children with hearing loss [14,19,24]. However, its impact on uptake, rather than timing alone, is poorly understood. Importantly, families face two key decision points in the CI referral pathway: (1) completing the CI evaluation after referral and (2) proceeding with implantation after candidacy determination. Understanding factors at each stage is essential to advancing equitable access to cochlear implantation.

We examined factors associated with family decision-making following pediatric CI referral, focusing on (1) the completion of the candidacy evaluation and (2) proceeding with cochlear implantation after candidacy determination. The primary aim was to identify contextual and audiologic factors that act as barriers to evaluation completion and CI uptake in children likely to benefit from CI use.

## 2. Materials and Methods

### 2.1. Data Acquisition

This study was approved by the University of North Carolina Chapel Hill Institutional Review Board (#22-0993). We queried our clinical database and identified children younger than 18 years who were referred for an initial CI evaluation between 1 January 2018 and 31 December 2024. Children who were determined not to be candidates at any point in the pathway were not included in this study, so the analyses reflected family choice among those eligible for implantation. This included (1) children referred but not evaluated because imaging identified cochlear nerve deficiency in the setting of single-sided deafness (SSD) and (2) children who completed evaluation but were not CI candidates because amplification (i.e., hearing aids) was deemed more appropriate than cochlear implantation.

The categorical outcome variable was referral disposition: (1) did not complete the CI candidacy evaluation (e.g., missed or refused appointments), (2) CI candidate who declined surgery, or (3) CI candidate who proceeded with surgery. Independent variables included county of residence, referral year (2018–2023), self-identified race/ethnicity per medical record (White, Black, Asian, Hispanic, mixed-race, or other), unaided hearing thresholds (ear-specific 4FPTA), insurance type (Medicaid or private), primary communication mode (spoken language, total communication, manual only, or not established), candidate type (traditional, residual hearing, AHL, or SSD), and age at referral (years).

County of residence was used to estimate socioeconomic status via the 2019 Social Deprivation Index (SDI). Originally developed by the Robert Graham Center, the SDI is a composite measure of socioeconomic disadvantage used to quantify socioeconomic variance in health outcomes. The SDI uses census-level data to create the measure, including the percentage of population living in poverty, average education level, percentage of single-parent households, home renting compared to ownership, percentage of population living in housing units defined as overcrowded, car ownership, and employment of senior citizens. The SDI is a scale from 1 to 100 with larger numbers representing higher levels of social deprivation [32]. For NC residents, rurality classifications were based on the NC Rural Center definitions [33]. Rural counties are classified as 250 people per square mile or less, regional cities and suburban counties with an average population density of 250–750 people per square mile, and urban counties of over 750 people per square mile [33]. Equivalent cutoffs were utilized for out-of-state residents.

Candidate types were defined as follows:

- Traditional: Bilateral severe-to-profound hearing loss.
- Non-traditional
  o Residual Hearing: Unaided thresholds ≤ 65 dB HL at 250 Hz.
  o AHL: Moderate-to-profound hearing loss in one ear and mild-to-moderately severe hearing loss contralaterally.
  o SSD: Normal hearing in one ear, moderate-to-profound hearing loss in the other

Patients are referred either by local community providers or by clinicians within the hospital system. Candidacy evaluations for CI are completed at the pediatric CI center, which is a separate clinic located approximately 4 miles from the nearest clinic housing physicians and diagnostic audiologists who also provide hearing aid fitting services.

A pediatric CI evaluation includes counseling about cochlear implantation and device options; a medical assessment with imaging (MRI and/or CT); a spoken-language evaluation by a listening and spoken language-certified speech–language pathologist; consultation with a social worker; and a comprehensive audiologic evaluation. Audiologic testing includes bilateral unaided air and bone conduction thresholds and the verification and validation of the hearing aid fitting. Verification is completed using the Desired Sensation Level (DSL) method with the measurement of the real-ear-to-coupler difference (RECD; [34,35]). Validation includes aided, ear-specific word recognition testing at 60 dB SPL for children with sufficient language to complete the task. For children who cannot complete speech perception testing, validation is completed using parent-reported and/or clinician-rated auditory skill measures, including LittlEars [36], the Functional Listening Index–Pediatric [37,38], and the Parents’ Evaluation of Aural/Oral Performance of Children (PEACH; [39]). For children with SSD, additional measures include the Speech, Spatial and Qualities (SSQ; [40]) questionnaire and the Vanderbilt Fatigue Scales [41].

Testing is typically conducted in English. Certified interpreters are used in person or via video as needed. Spanish-language speech and language evaluations are always completed with an in-person interpreter. CI candidacy is determined through multidisciplinary team discussion, after which families are counseled on results and recommendations.

### 2.2. Data Analysis

Binomial logistic regression models were used to examine the predictors of (1) CI evaluation completion and (2) CI uptake among candidates. Models were constructed using the glmm [42] function v3.1-66 in R Studio 2023.06.1 [43]. The same independent variables were entered into both models: county of residence, referral year, race/ethnicity, unaided hearing thresholds, insurance type, primary communication mode, candidate type, and age at referral.

## 3. Results

Descriptive statistics are listed in Table 1 and Table 2. The sample consisted of 719 patients. The average patient was male (*n* = 373, 52%) and White (*n* = 365, 51%) and resided in a rural area (*n* = 294, 41%). The racial and ethnic composition of the sample was slightly more diverse than that reported in census data (see Table 1; [44]). The mean SDI was 0.01 (SD = 0.81), mean age at referral was 6.1 years (SD = 5.0), and mean poorer-ear 4FPTA was 94.6 dB HL (SD = 20.0). Overall, 112 patients (16%) did not complete a CI evaluation, 109 (15%) were determined to be CI candidates but declined cochlear implantation, and 498 children (69%) were determined to be CI candidates and proceeded with cochlear implantation.

### 3.1. Model 1: Predictors of Evaluation Completion

**Figure 1:**
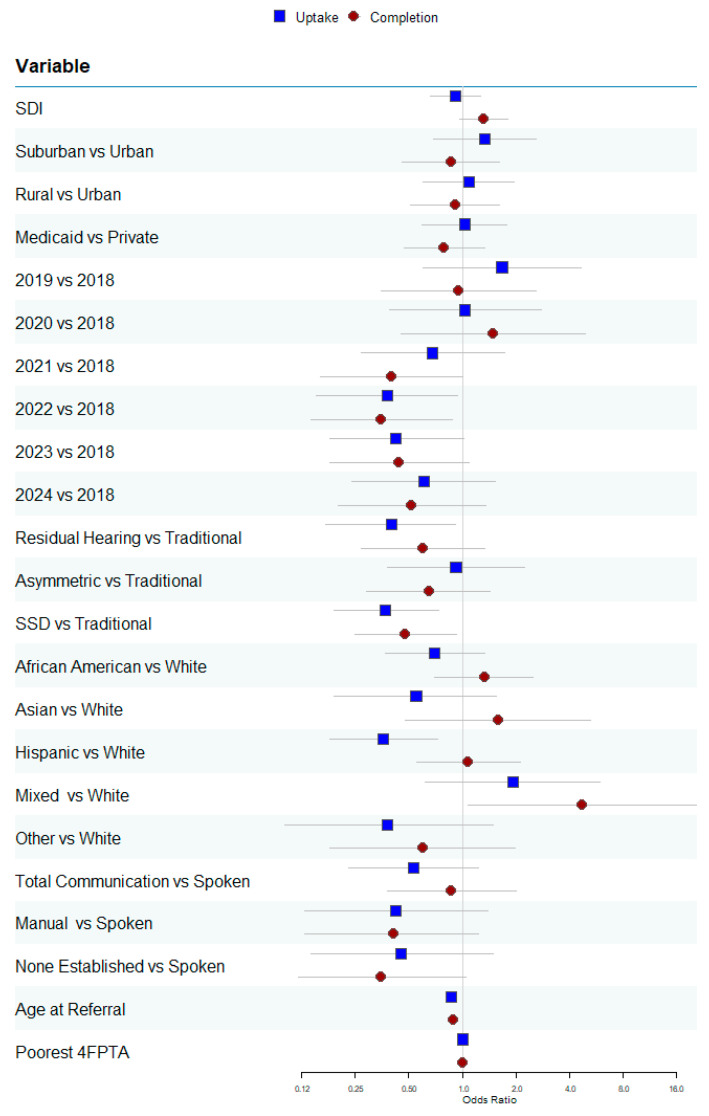
Odds ratios and 95% confidence intervals for each variable. Findings for referral follow-through are in red circles, and findings for cochlear implant uptake are in blue squares.

**Figure 2:**
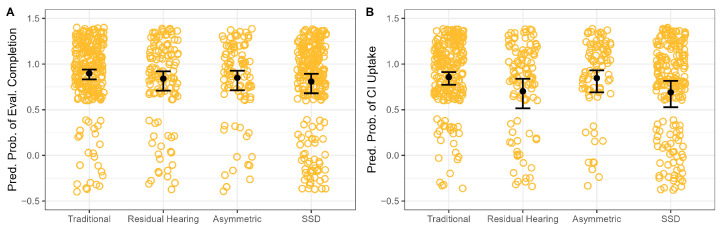
Distribution of outcome probability based on candidate type. Panel (**A**) shows referral follow-through, and (**B**) shows uptake among candidates. Circles represent individual patients. Error bars represent 95% confidence interval.

**Figure 3:**
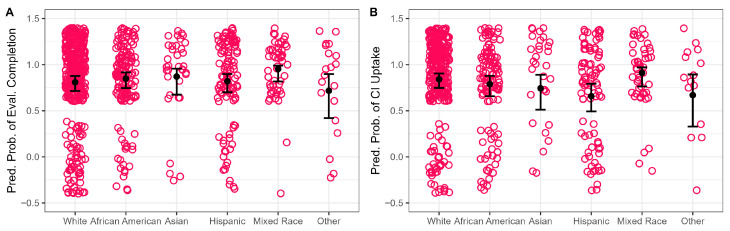
Distribution of outcome probability based on race/ethnicity. Panel (**A**) shows referral follow-through, and (**B**) shows uptake among candidates. Circles represent individual patients. Error bars represent 95% confidence interval.

**Figure 4:**
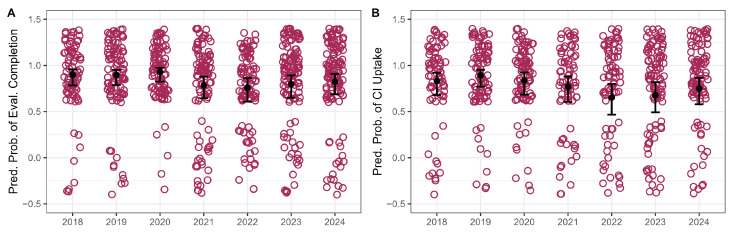
Distribution of outcome probability based on year of referral. Panel (**A**) shows referral follow-through, and (**B**) shows uptake among candidates. Circles represent individual patients. Error bars represent 95% confidence interval.

**Figure 5:**
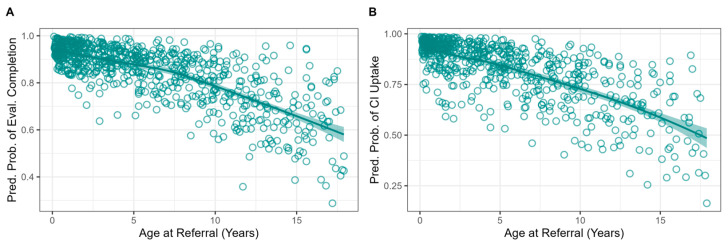
Regression showing outcome probability based on age at referral. Panel (**A**) shows referral follow-through, and (**B**) shows uptake among candidates. Circles represent individual patients. Shaded area represents 95% confidence interval.

The regression results are presented in Table 3 and plotted in Figure 1, Figure 2, Figure 3, Figure 4 and Figure 5. Of the 719 children referred for CI evaluation, 84% completed the process. The completion of the evaluation was not significantly associated with the SDI (*p* = 0.089), insurance type (*p* = 0.382), rurality (*p* = 0.651), or 4FPTA in the poorer ear (*p* = 0.579). There was a trend toward lower referral adherence among children without an established communication mode compared with those using spoken language, although this did not reach statistical significance (*p* = 0.059). Significant predictors of evaluation completion included candidate type, race/ethnicity, year of referral, and age (see Table 3).

Compared with traditional candidates, children with SSD were significantly less likely to complete a CI evaluation (80% vs. 90%), with about 52% lower odds of completion (OR ≈ 0.48, *p* = 0.029). No significant differences in evaluation completion were observed for candidates with residual hearing (82%, *p* = 0.215) or asymmetric hearing loss 84% (*p* = 0.281).

Children identified as mixed-race were significantly more likely than White children to complete the evaluation (96% vs. 84%; *p* = 0.041), with about 4.6× higher odds (OR ≈ 4.69). No significant differences were observed between White children and those identifying as African American (85%), Asian (89%), Hispanic (81%), or other (75%; all *p* > 0.375).

Evaluation follow-through varied by year of referral. In the reference year (2018), 90% of referred children completed the evaluation. Completion rates decreased to 75% in 2021 (*p* = 0.053), with about 60% lower odds than 2018 (OR ≈ 0.40), and declined significantly to 77% in 2022 (*p* = 0.026), with about 65% lower odds (OR ≈ 0.35). Evaluation completion rates in 2019 (89%), 2020 (95%), 2023 (81%), and 2024 (85%) did not differ significantly from that in 2018.

Age at referral was a significant predictor of evaluation completion (*p* < 0.001), with younger children more likely to complete the evaluation. Children who completed the evaluation had a mean age of 5.5 years (SD = 4.8), whereas those who did not had a mean age of 9.1 years (SD = 5.1). Each one-year increase in age was associated with an 11% decrease in the likelihood of evaluation completion (OR ≈ 0.89).

### 3.2. Model 2: Predictors of CI Uptake

The regression results are summarized in Table 4 and plotted in Figure 1, Figure 2, Figure 3, Figure 4 and Figure 5. Among CI candidates (*n* = 607), 82% proceeded with CI surgery. Non-significant predictors of uptake were the SDI (*p* = 0.574), insurance type (*p* = 0.930), rurality (*p* = 0.384), communication mode (*p* = 0.142), and poorer-ear 4FPTA (*p* = 0.870). Candidate type, race/ethnicity, year of referral, and age were significant predictors (see Table 4).

Uptake was the highest among traditional candidates (89%). It was significantly lower for candidates with residual hearing (77%; *p* = 0.031) and for those with SSD (74%; *p* = 0.005), corresponding to 60% and 63% lower odds of uptake, respectively (OR ≈ 0.40 and 0.37). Uptake among candidates with asymmetric hearing loss (87%; *p* = 0.858) did not differ significantly from that of traditional candidates.

Children who identified as Hispanic were significantly less likely to undergo implantation than non-Hispanic White children (69% vs. 87%; *p* = 0.004), at 64% lower odds (OR ≈ 0.36). Uptake among children identified as African American (77%), Asian (77%), mixed-race (92%), or other (77%) did not differ significantly from that of White children (all *p* > 0.261).

In 2018, 85% of candidates proceeded with implantation. Uptake declined to 73% in 2022 (*p* = 0.036), corresponding to 62% lower odds of uptake than in 2018 (OR ≈ 0.38). A decline was also observed in 2023 (75% uptake; OR ≈ 0.42, 58% lower odds), although this difference did not reach statistical significance (*p* = 0.055). The rates of change in other years were not significantly different (all *p* > 0.293).

Age at referral was also a significant predictor of CI uptake (*p* < 0.001). Children who proceeded with cochlear implantation were younger (M = 4.9 years; SD = 4.5) than those who did not proceed with cochlear implantation (M = 8.4 years; SD = 4.8). Each additional year of age was associated with 13% lower odds of uptake (OR ≈ 0.87).

## 4. Discussion

This study identified factors predictive of pediatric CI evaluation completion and CI uptake, underscoring that barriers to CI care emerge at multiple points along the referral pathway. Candidate type, race and ethnicity, year of referral, and age at referral were predictive of decision-making, though their effects varied by stage. For example, children with SSD were less likely to complete CI evaluations, and CI candidates with SSD or residual hearing were less likely to proceed with implantation. Differences by race and ethnicity also varied by stage, with higher evaluation completion among children identified as mixed-race and lower implantation uptake among children identified as Hispanic. Temporal patterns further distinguished these outcomes, with evaluation follow-through beginning to decline in 2021, significant reductions in both evaluation completion and implantation uptake in 2022, and continued lower uptake in 2023, consistent with delayed downstream effects in the referral pathway. Younger age at referral was associated with an increased likelihood of both evaluation completion and CI uptake. In contrast, the SDI, rurality, degree of hearing loss, insurance source, and communication mode were not significantly associated with either outcome.

### 4.1. Candidate Type and Decision-Making

Candidate type emerged as a factor influencing decision-making at multiple points in the pediatric CI care pathway. Children with SSD were less likely to complete CI evaluations following referral, and both children with SSD and those with residual hearing were less likely to proceed with implantation once candidacy was established. These findings align with prior work demonstrating greater variability in referral patterns and uptake among non-traditional CI candidates, particularly those with SSD and residual hearing [10,21,23].

One potential explanation for these findings relates to perceived functional impact and motivation for intervention. Compared with traditional and asymmetric hearing loss candidates, children with SSD or residual hearing have more unaided acoustic access, which may reduce the perceived impact of hearing loss. In this context, academic challenges, fatigue, or attentional concerns may be attributed to factors other than hearing loss, potentially reducing urgency for intervention. For children with SSD who have not used amplification, cochlear implantation may also represent a transition from an “invisible” to a visible disability, which has been described in the prior literature as a potential concern related to device visibility, stigma, and the societal perceptions of disability and may influence family decision-making [45].

Lower evaluation completion among children with SSD may reflect variability in referral counseling. Although growing evidence supports CI use in pediatric SSD [6,9,46,47,48,49,50,51,52], families may encounter variability in counseling or messaging regarding expected benefit, the role of the timing of intervention, or the necessity of implantation when one ear has typical hearing [46,53]. This uncertainty may contribute to lower referral follow-through.

Similarly, reduced CI uptake among children with residual hearing highlights the complexity of surgical decision-making in this population. Families may weigh anticipated benefits against concerns regarding surgical risk or the loss of residual acoustic hearing, particularly when functional benefit with hearing aids is present [54].

Together, these findings suggest that expanding candidacy alone may be insufficient to ensure equitable uptake across hearing loss configurations and underscore the need for configuration-specific counseling strategies.

### 4.2. Race, Ethnicity, and Health Equity Considerations

The differences in evaluation completion and implantation uptake by race and ethnicity observed in this study are consistent with the broader hearing health equity literature [30,55,56,57,58,59]. Notably, commonly used proxies for structural disadvantage (e.g., SDI, insurance type, rurality) were not significant predictors in this cohort, suggesting that these proxy measures may not fully capture more nuanced factors influencing engagement and care. Prior work by Schuh and Bush discusses barriers such as language, cultural differences, and household income. Neither the SDI, insurance type, nor rurality directly measure these barriers. These findings should not be interpreted solely as differences in family preferences or decision-making but rather as potential indicators of differential exposure to structural, cultural, and healthcare system-level factors that influence engagement with providers [60,61,62].

In this sample, children who identified as mixed-race were more likely to complete CI evaluations, whereas children who identified as Hispanic were less likely to proceed with implantation once candidacy was established. The variance in these findings across the stages of the CI pathway suggests that factors supporting referral follow-through may differ from those influencing decisions about surgical intervention. Race in this context should be understood as a proxy for lived experience within healthcare systems, rather than a biological determinant, and observed differences may reflect variation in access, support, and navigation across the care pathway [63].

The observed association for mixed-race children should be interpreted with caution. Although this group was adequately represented relative to other subgroups, the sample size remains smaller than that for White and African American children. This finding may reflect unmeasured differences in care navigation, prior healthcare engagement, or referral patterns rather than a direct effect of racial identity itself. Prior work in pediatric specialty care has demonstrated that families from historically marginalized racial and ethnic groups may encounter increasing barriers later in care trajectories, including language discordance, limited access to culturally responsive counseling, and challenges related to informed consent for invasive procedures [17,26,62].

Lower implantation uptake among children who identified as Hispanic aligns with previous studies documenting disparities in access to cochlear implantation [10,25]. Potential contributors described in the prior literature include language barriers, the limited availability of interpretation services, variability in provider counseling, and broader structural factors such as immigration-related concerns or mistrust rooted in prior healthcare experiences [64,65,66]. These mechanisms were not directly measured in the present study, however, and should be interpreted as plausible explanations rather than confirmed drivers of the findings. Together, these findings underscore that disparities in CI care are not uniform across the care continuum and likely reflect distinct structural and contextual influences at each stage.

### 4.3. Temporal Trends and COVID-19-Era Effects

Year of referral was a significant predictor of both CI evaluation completion and CI uptake, though effects differed slightly. Evaluation completion showed a non-significant decline in 2021, followed by significant reductions in both evaluation completion and implantation uptake in 2022, with uptake remaining lower in 2023. This pattern of results needs cautious interpretation. Year of referral is an imperfect proxy for when care was delivered as there are commonly delays between identification, referral, evaluation, and implantation. Hence, the outcomes associated with a referral year may reflect both the concurrent and lingering effects of interruptions in healthcare.

These findings are consistent with, but not definitive of, COVID-19-related disruptions in care. Pandemic-related disruptions, including reduced access to specialty services, delays in non-urgent care, and increased logistical and psychosocial stressors for families, may have impacted earlier stages of the referral pathway, resulting in downstream effects on evaluation completion and surgical uptake emerging in subsequent years [67,68,69,70]. While rates in later years appear to improve, uptake in 2023 remained below pre-pandemic levels, suggesting partial but not complete recovery. These findings emphasize the need for flexible, resilient care models that can maintain continuity and support informed decision-making.

### 4.4. Age at Referral

Age at referral was a predictor of both CI evaluation completion and implantation uptake, with younger children more likely to progress through each stage of care. Lower follow-through rates among older children may reflect factors such as reduced perceived need and urgency, greater child autonomy in decision-making, and/or fear of surgery [71,72,73]. Older children may also have longer or less clearly defined durations of deafness, an important prognostic factor discussed during CI counseling and associated with reduced expected auditory outcomes. In contrast, the families of younger children often receive consistent messaging emphasizing early intervention due to its importance for auditory development.

### 4.5. Clinical and System-Level Implications

Several factors commonly associated with disparities in pediatric hearing healthcare, including socioeconomic deprivation, rural residence, insurance source, degree of hearing loss, and communication mode, were not significantly associated with evaluation completion or CI uptake in this cohort. These findings may reflect the partial mitigation of structural barriers through centralized specialty care models, particularly in a state such as NC, with a limited number of pediatric CI centers accepting Medicaid. However, the presence of significant influences of race/ethnicity and candidate type suggests that disparities persist through mechanisms not captured by variables focused on access.

Distinct predictors for candidacy evaluation completion and CI uptake underscore the importance of viewing CI uptake as a multi-stage process, with different challenges emerging at each decision point. Referral alone is insufficient to ensure access to cochlear implantation. Clinically, these results highlight the need for counseling strategies tailored to candidate type, age at referral, and family context. For example, children with SSD or residual hearing may benefit from counseling that directly addresses perceived necessity, expected benefit, and concerns related to the preservation of residual hearing, while the families of older children may require discussions focused on functional, educational, and psychosocial outcomes. Targeted, equity-focused interventions, including culturally and linguistically responsive counseling, structured follow-up after referral, and flexible care models, are needed to support families through evaluation and implantation decisions and to ensure that expanded candidacy translates into equitable access for pediatric patients.

### 4.6. Limitations and Future Directions

The retrospective design of this study limits the ability to infer causality and relies on the accuracy and completeness of information documented in a clinical database. Decisions to defer CI evaluation or decline implantation were deduced from documented follow-through rather than directly reported from families.

The information presented in this dataset may not fully capture factors that impact decision-making at each stage of the care pathway. Variables such as parental education level, language proficiency, cultural beliefs towards medical intervention and surgery, and prior history with hearing devices are examples of speculative factors cited as barriers to optimal hearing care and were not available for this study [14,16,24,26,28,58]. Their absence may introduce bias in interpreting these findings. For example, families with higher education levels may be better able to navigate referral processes, schedule and attend specialty appointments, and engage in complex counseling discussions. This could increase the likelihood of completing a CI evaluation. Similarly, children with consistent prior hearing aid use may represent families already engaged in hearing healthcare, making them more likely to follow through with evaluation and treatment recommendations. These factors are not randomly distributed across patient populations and may systematically advantage or disadvantage families at different stages of care. The interpretations of the underlying mechanisms discussed in the current work should be viewed as hypothesis-generating and grounded in the prior literature, rather than directly tested within this dataset.

Additionally, this study was conducted at a single pediatric CI center, which may limit generalizability to other regions and clinical settings. As one of the few CI centers in NC that accepts all types of Medicaid, patients who face greater financial and systemic barriers to care may be overrepresented relative to centers that only accept private insurance. Full participation in Medicaid at our center may in turn bias factors such as the SDI and rurality with families traveling larger distances to receive care covered through insurance. Conversely, CI evaluation at this center is completed by a different set of providers in a different location than their familiar hearing healthcare environment. Some children stay within the university system, and others are establishing care with an entirely different clinic. These factors may influence evaluation completion and CI uptake and were not included in the current study. Additionally, 80% of children in this study were identified as utilizing or learning spoken language. With most referrals consisting of families focusing on spoken language development, it is unsurprising that communication mode was not found to be significant. A recent study by Campbell and Bergelson [74] evaluated language progress in deaf and hard-of-hearing toddlers enrolled in early intervention in NC. This study noted that no participants utilized sign language and cited the state’s lack of resources for families to learn American Sign Language. North Carolina is known to focus on listening and spoken language [74,75]. This may limit generalizability areas with greater exposure to signed languages.

Future research should incorporate prospective and mixed-methods approaches to better characterize family decision-making throughout the CI referral pathway. Qualitative studies involving caregivers and providers may provide critical insight into perceived barriers, informational needs, and points of disengagement, particularly among the families of non-traditional CI candidates and those from historically marginalized communities. Multisite studies are also needed to evaluate whether the patterns observed here persist across different healthcare systems and geographic contexts.

## 5. Conclusions

Together, these findings highlight that pediatric CI access is shaped by stage-specific factors along the referral pathway and underscore the need for targeted strategies to support families from initial referral to implantation.

## Figures and Tables

**Table 1:** Categorical subject details.

Category	Factor	*n*	% of Total Sample
Communication Mode	Spoken Language	583	81%
	Total Communication	70	10%
	None Established	36	5%
	Manual	30	4%
Race/Ethnicity	White (NC population %)	365	51 (61%)
	African American (NC population %)	140	20 (20%)
	Hispanic (NC population %)	109	15 (11%)
	Mixed Race (NC population %)	50	7 (4%)
	Asian (NC population %)	35	5 (3%)
	Other (NC population %)	20	3 (1%)
Insurance	Private	225	48%
	Medicaid	233	50%
Candidate Type	Traditional	250	35%
	SSD	247	34%
	Residual Hearing	124	17%
	AHL	98	14%
Year of Referral	2023	124	17%
	2024	121	17%
	2019	101	14%
	2021	100	14%
	2022	97	14%
	2020	95	13%
	2018	81	11%
Rurality	Rural	294	41%
	Urban	247	34%
	Suburban	178	25%

AHL, asymmetric hearing loss; SSD, single-sided deafness.

**Table 2:** Continuous variable patient details.

Factor	Mean	SD
Social Deprivation Index	0.01	0.81
Poorest-Ear 4FPTA (dB HL)	94.6	20.0
Age at Referral (Years)	6.1	5.0

Note: 4FPTA, four-frequency pure-tone average (500, 1000, 2000, and 4000 Hz threshold average).

**Table 3:** Results of binomial logistic regression investigating factors influencing completion of cochlear implant evaluation following referral. Comparison variable is in brackets.

Category	Predictors	Estimates	SE	*p*
(Intercept)	Completed [Not Completed]	3.405	0.823	<0.001
SDI		0.271	0.60	0.089
Age at Referral		−0.121	0.023	<0.001
Poorer-Ear 4FPTA (dB HL)		0.003	0.006	0.578
Insurance	Public [Private]	−0.231	0.265	0.382
Rurality	Suburban [Urban]	−0.144	0.319	0.651
	Rural [Urban]	−0.097	0.293	0.742
Year	2019 [2018]	−0.51	0.513	0.921
	2020 [2018]	0.401	0.609	0.510
	2021 [2018]	−0.905	0.467	0.053
	2022 [2018]	−1.049	0.470	0.026
	2023 [2018]	−0.821	0.465	0.077
	2024 [2018]	−0.646	0.486	0.183
Candidate Type	Residual Hearing [Traditional]	−0.504	0.406	0.215
	AHL [Traditional]	−0.438	0.407	0.281
	SSD [Traditional]	−0.731	0.335	0.029
Race/Ethnicity	African American [White]	0.386	0.322	0.375
	Asian [White]	0.465	0.607	0.443
	Hispanic [White]	0.073	0.344	0.832
	Mixed Race [White]	1.546	0.756	0.041
	Other [White]	−0.511	0.608	0.401
Communication Mode	Total Communication [Spoken]	−0.134	0.424	0.753
	Manual [Spoken]	−0.899	0.564	0.111
	None Established [Spoken]	−1.60	0.652	0.159
	*N*	719		
	Pseudo R^2^ (Nagelkerke)	0.196		

Note: 4FPTA, pure-tone average at 500, 1000, 2000, and 4000 Hz; AHL, asymmetric hearing loss; SDI, social deprivation index; SE, standard error; SSD, single-sided deafness.

**Table 4:** Results of binomial logistic regression investigating factors influencing cochlear implant uptake among pediatric candidates. Comparison variable is in brackets.

Category	Predictors	Estimates	SE	*p*
(Intercept)	Completed [Not Completed]	3.776	0.887	<0.001
SDI		−0.093	0.165	0.574
Age at Referral		−0.136	0.025	<0.001
Poorest-Ear 4FPTA (dB HL)		−0.001	0.007	0.870
Insurance	Public [Private]	0.025	0.282	0.930
Rurality	Suburban [Urban]	0.293	0.337	0.384
	Rural [Urban]	0.082	0.301	0.786
Year	2019 [2018]	0.514	0.520	0.322
	2020 [2018]	0.033	0.503	0.947
	2021 [2018]	−0.388	0.479	0.418
	2022 [2018]	−0.967	0.461	0.036
	2023 [2018]	−0.856	0.445	0.055
	2024 [2018]	−0.498	0.474	0.293
Candidate Type	Residual Hearing [Traditional]	−0.926	0.428	0.030
	AHL [Traditional]	−0.081	0.450	0.858
	SSD [Traditional]	−0.983	0.347	0.005
Race/Ethnicity	African American [White]	−0.351	0.328	0.284
	Asian [White]	−0.602	0.535	0.261
	Hispanic [White]	−1.015	0.357	0.004
	Mixed Race [White]	0.651	0.579	0.261
	Other [White]	−0.962	0.695	0.166
Communication Mode	Total Communication [Spoken]	−0.628	0.428	0.142
	Manual [Spoken]	−0.874	0.614	0.155
	None Established [Spoken]	−0.793	0.606	0.190
	*N*	607		
	Pseudo R^2^ (Nagelkerke)	0.226		

AHL, Asymmetric Hearing Loss.

## Data Availability

The data presented in this study are available on request from the corresponding author due to IRB restrictions.

## Abbreviations

The following abbreviations are used in this manuscript:

4FPTA	Four-Frequency Pure-Tone Average
AHL	Asymmetric Hearing Loss
CI	Cochlear Implant
FDA	Food and Drug Administration
M	Mean
NC	North Carolina
SD	Standard Deviation
SDI	Social Deprivation Index
SSD	Single-Sided Deafness
US	United States
